# Doxorubicin-loaded PEG-CdTe QDs conjugated with anti-CXCR4 mAbs: a novel delivery system for extramedullary multiple myeloma treatment

**DOI:** 10.1007/s10856-023-06772-w

**Published:** 2024-01-20

**Authors:** Dangui Chen, Fei Chen, Jia Lu, Lihong Wang, Fusheng Yao, Haitao Xu

**Affiliations:** Department of hematology, Anqing Municipal Hospital, Anqing, 246003 People’s Republic of China

## Abstract

**Abstract:**

Extramedullary multiple myeloma (EMM) is defined as the presence of plasma cells outside the bone marrow of multiple myeloma patients, and its prognosis is poor. High-dose chemotherapy with autologous stem cell transplantation, as a good option on early lines of therapy, has retained the survival benefit of youny EMM patients, but is intolerant for the majority of old patients because of drug cytotoxicity. To essentially address the intolerance above, we designed a CXCR4-PEG-CdTe-DOX (where CXCR4: chemokine receptor 4; PEG-CdTe: polyethylene glycol-modified cadmium telluride; DOX:doxorubicin) nanoplatform. First, CXCR4 is highly expressed in extramedullary plasma cells. Second, PEG-CdTe a drug carrier that controls drug release, can reduce adverse reactions, prolong drug (e.g, DOX) circulation time in the body, and form a targeting carrier after connecting antibodies. In vitro experiments showed CXCR4-PEG-CdTe-DOX facilitated intracellular drug accumulation through active CXCR4 targeting and released DOX into the microenvironment in a pH-controlled manner, enhancing the therapeutic efficacy and apoptosis rate of myeloma cells (U266). Therefore, targeted chemotherapy mediated by CXCR4-PEG-CdTe-DOX is a promising option for EMM treatment.

**Graphical abstract:**

**Figure:**
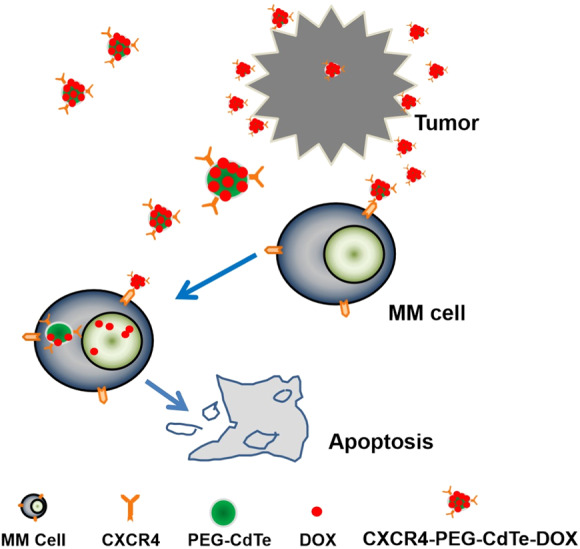
Figure 1 Action mechanism of drug delivery pathway: CXCR4-PEG-CdTe-DOX nanocomplex can be targeted and close to MM cell membranes, effectively transport DOX into MM cells, and promote MM cell apoptosis.

## Introduction

Extramedullary multiple myeloma (EMM) is a rare but recognized manifestation of multiple myeloma (MM) and is characterized by the proliferation of malignant plasma cells outside the confines of bone marrow. EMM can occur not only upon the diagnosis of MM (3–5%) but also at the time of relapse (up to 28%) [1, 2]. The survivability of MM patients has improved significantly over the past decades, and the recent estimated 5-year overall survival (OS) rate is 70% among the transplant-eligible patients and 50% in the elderly [2]. In a study that strictly restricts the definition of EMM to biopsy-proven EMM, the median OS from the time of extramedullary diagnosis is 1.3 years [3]. However, extramedullary recurrence will develop and the median time to patient survival is less 6 months [4].

Extramedullary diseases are a hallmark for the worse prognosis of MM patients, regardless of other high-risk factors. Bortezomib-based therapy does not improve the survival of EMM patients, but high-dose of chemotherapy reportedly can improve their prognosis [5]. In a retrospective series, the median progression-free survival and the OS of primary EMM patients undergoing autologous stem cell transplantation (ASCT) are 38.9 and 46.5 months respectively [6]. Otherwhile, MM is frequently seen in elderly patients, with a median onset age of 69 years old [7]. Elderly patients are often combined with organ damage diseases, and generally cannot receive intensive chemotherapy or ASCT.

Chemotherapy is still the main treatment for EMM, but high-dose chemotherapy inevitably causes damages, even irreversible damages, to normal tissues and organs. Luckily, the emerging new drugs (e.g., proteasome inhibitors and immunomodulators) has improved the remission rate and survival time of MM patients, but almost all patients will relapse [8]. Hence, MM is still an incurable disease. In high-dose chemotherapy, the synergistic effect of anthracyclines (e.g., doxorubicin) combined with new drugs (e.g., proteasome inhibitors) can improve the remission rate and prolong the survival time of EMM patients. However, DOX increases serious adverse events in EMM patients.

Despite the significantly prolonged survival time owing to the emergence of new drugs in recent decades, MM is still an incurable disease with short survival and poor prognosis, especially for EMM patients. Hence, the treatment of EMM still needs to be explored.

Reportedly, chemokine receptor is overexpressed in various malignancies, which suggests a poor prognostic factor [9]. In addition, CXCR4 receptor analogs showe a good imaging diagnosis in the detection of extramedullary lesions among MM patients [10]. Mature B lymphocytes are located outside the pulp, and secreted plasma cells can migrate to the pulp cavity through the SDF1/CXCR4 pathway, playing an important role in the homing and migration of MM cells. The SDF-1/CXCR4 axis is involved in the proliferation, angiogenesis, anti-apoptosis and metastasis of tumor cells [11]. CXCR4 is overexpressed in extramedullary myeloma cells and associated with the poor prognosis of extramedullary myeloma patients [12, 13]. [68Ga]Pentixafor(CXCR4 antagonist)-PET/CT can improve the detection rate of extramedullary lesions in MM patients compared with [18 F]FDG-PET/CT [14], and [68Ga]Pentixafor-PET/CT. Furthermore, CXCR4 is often expressed in the extramedullary lesions of MM patients. Thus, CXCR4 is considered a potential drug target.

Tumor cell migration and settlement are the fundamental pathways of extramedullary infiltration. Thus, it is reasonable to believe that targeting CXCR4 can antagonize the combination of CXCR4 and SDF-1, remove tumor cells out of the tissue site, and make them more accessible to traditional treatments. This strategy also can target and eliminate CXCR4 myeloma cells, and fundamentally block the migration and extramedullary infiltration of tumor cells.

**Fig. 1:**
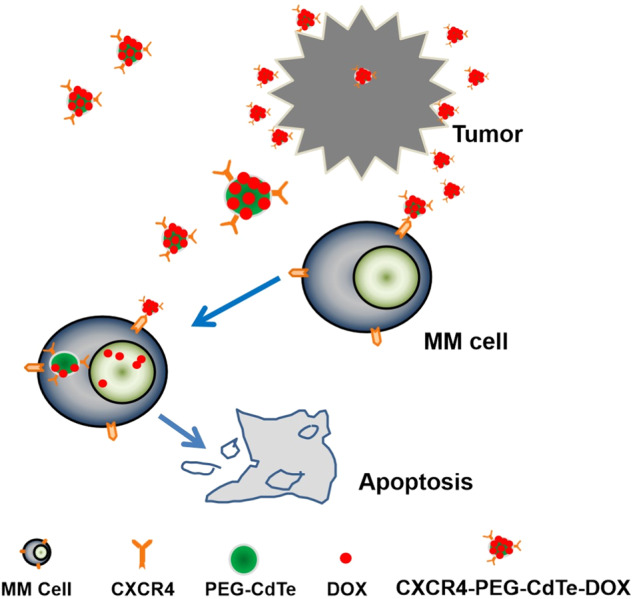
Action mechanism of drug delivery pathway: CXCR4-PEG-CdTe-DOX nanocomplex can be targeted and close to MM cell membranes, effectively transport DOX into MM cells, and promote MM cell apoptosis

Here, we designed a CXCR4-PEG-CdTe-DOX nanoplatform that can essentially address the challenge above. Specifically, it is an active targeted drug delivery system that actively delivers DOX to U266 cells and improves chemotherapeutic efficacy. The schematic map is shown in Fig. 1. This system can significantly improve the antitumor effect of DOX in U266 MM cells in vitro. We expect this strategy will throw light onto clinical cancer treatment.

## Materials and methods

### Materials

PEG-CdTe QDs and dimethyl sulfoxide (DMSO) were purchased from Sigma-Aldrich (St. Louis, USA). DOX was obtained from Sigma (Wuhan, China). 1-(3-dimethylamino propyl)-3-ethyl carbon diimine hydrochloride (EDC) and N-hydroxysuccinide (sulfo-NHS) were offered by Sigma(Shanghai,China). Roswell Park Memorial Institute medium (RPMI) 1640 and fetal bovine serum (FBS) were bought from Gibco Chemical Co. (Carlsbad, CA, USA). Pierce™ bicinchoninic acid (BCA) protein assay kits, Annexin V-fluorescein isothiocyanate (FITC) apoptosis detection kits, and hematoxylin–eosin kits were provided by KeyGen Biotech Co., Ltd. (Jiangsu, China). Cell counting kit-8 (CCK-8) was obtained from Dojindo Molecular Technologies. Inc. (Nanjing, China). Monoclonal antibodies for CXCR4, SURVIVIN, Bcl-xl, cleaved caspase-3/9, and glyceraldehyde-3-phosphate dehydrogenase (GAPDH) were made in Santa Cruz Biotechnology Inc. (CA, USA). All reagents were analytically pure.

### Synthesis and characterization of CXCR4-PEG-CdTe-DOX [8]

The anti-CXCR4 monoclonal antibodies were coupled to the PEG-CdTe QDs (PEG is ended with carboxyl group) using EDC/NHS methods [15]. The drug-loading procedure is shouwn below. First, PEG-CdTe QDs, EDC and NHS (each 1 mg) were dispersed separately into 1 mL of deionized water, forming 1 mg/mL solutions. Then, 1 mL of PEG-CdTe QDs, 0.1 mL of EDC and 0.1 mL of sulfo-NHS were mixed and stirred for 1 h at room temperature in the dark, Next, 10 μL of anti-CXCR4 monoclonal antibodies (1 mg/mL) was stirred with carboxyl-activated PEG-CdTe QDs for 1 h in the dark under shaking at 100 rpm and 37 °C and connected via amide bonds. The anti-CXCR4-coupled PEG-CdTe QDs were purified after 30 min of centrifugation at 15,000 rpm.

DOX can be efficiently absorbed onto the CXCR4-PEG-CdTe QDs through electrostatic interaction [8]. DOX (1 mg/mL, 1 mL) was added to 1 mL of CXCR4-PEG-CdTe (1 mg/mL) under stirring for 6 h in the dark. CXCR4-PEG-CdTe-DOX was formed after stepwise procedures, including centrifugation, removal of supernatant, washing, precipitation, and resuspension. The unbound DOX in the supernatant was detected with high-performance liquid chromatography (HPLC). Then Drug loading (DL) and Encapsulation efficiency (EE) were computed as follows: DL = (Amount of drug in NPs / Amount of NPs) × 100%; EE = (Amount of drug in NPs / Amount of the total drug) × 100% (where NPs: nanoparticles). The morphological properties of the nanoparticles were observed under transmission electron microscopy (TEM). Hydrodynamic diameters, Zeta potentials and size distributions of both PEG-CdTe, PEG-CdTe-DOX and CXCR4-PEG-CdTe-DOX were measured via dynamic light scattering (DLS). Meanwhile, the protein bands of PEG-CdTe QDs, anti-CXCR4 monoclonal antibodies and CXCR4-PEG-CdTe QDs were stained with Coomassie brilliant blue R250, after gelelectrophoresis, to determine whether the anti-CXCR4 monoclonal antibodies were coupled to PEG-CdTe.

### In vitro Release from CXCR4-PEG-CdTe-DOX

According to our previous study [8], the release of DOX from CXCR4-PEG-CdTe-DOX was measured at pH 6.0 (typical pH of peri-tumor environment) and pH 7.4 (typical pH of normal tissue microenvironment). CXCR4-PEG-CdTe-DOX (50 mL, DOX 0.1 mg/mL) was enclosed in dialysis bags, which were then immersed in 100 mL of phosphate-buffered saline (PBS) under continuous shaking (100 rpm) at 37 °C. Aliquots (0.1 mL) were removed from the PBS at a predetermined time interval and compensated with an equivalent volume of fresh PBS each time. The DOX concentration was quantified with HPLC.

### Cell culture

U266 cells, an MM cells line, were bought from Shanghai Institute of Cells (China). The U266 cells were cultured at 37 °C with 5% CO_2_ in RPMI 1640, which was supplemented with 10% FBS, 100 U/mL penicillin, and 100 μg/mL streptomycin. The medium was renewed every 2–3 days to maintain the best state.

### Cellular uptake

Briefly, the U266 cells were incubated for 3 h with control, DOX, PEG-CdTe-DOX or CXCR4-PEG-CdTe-DOX. Then these cells were collected via centrifugation and 800 rpm for 5 min. The culture medium was discarded and the precipitate was dispersed in 400 μL of PBS. Finally, cellular uptake was analyzed via flow cytometry (FCM). The relative fluorescence intensity of DOX was calculated as: count of FI-treated cells / count of FI control cells.

### Cell viability assay

The cell viability of U266 cells was measured using CCK-8 to estimate the cytotoxicity of PEG-CdTe, DOX, PEG-CdTe-DOX or CXCR4-PEG-CdTe-DOX. The relative concentration for PEG-CdTe was 0.1–1.6 ug/mL. After 24 h of treatment, each well was added with 10 μL of a CCK-8 solution and incubated for another 4 h. Then the optical density (OD) of the wells at 450 nm was read in a microplate reader. In another experiment, a 96-well plate was seeded with U266 cells at a concentration of 5 × 10^4^ /cells. Then the U266 cells were treated with PBS (as the control group), CXCR4, PEG-CdTe, DOX, PEG-CdTe-DOX or CXCR4-PEG-CdTe-DOX. The DOX concentration was 0.979 ug/mL in different forms. Each group was tested in triplicate. After incubation for 24, 48, or 72 h at 37 °C in a humidified atmosphere with 5% CO_2_, each well was added with 10 μL of a CCK-8 solution and incubated for another 4 h with the same method described above. Growth inhibition rates (%) of the U266 cells were calculated as (1-OD_treatment_/OD_control_) × 100.

### Cell targeting

U266 cells (5 × 10^4^/mL) were exposed to FITC-CXCR4-PEG-CdTe-DOX for 2 h. Subsequently, the U266 cells were washed with cold PBS, and the cell cultures were drop-cast on a clean glass slide. The samples were photographed with a confocal inverted microscope. The emission wavelengths of FITC and DOX were 550 and 625 nm, respectively. All optical measurements were implemented at room temperature (25 ± 2 °C).

### Cell apoptosis experiment

The U266 cells (5 × 10^4^/mL) were exposed to the control, CXCR4, PEG-CdTe, DOX, PEG-CdTe-DOX or CXCR4-PEG-CdTe-DOX for 24 h. Afterwards, the cells were washed with cold PBS, and stained with 5 μL of Annexin V-FITC and 5 μL of propidium iodide (PI). Cell apoptosis was quantified with FCM.

### Western blotting

U266 cells were harvested after different treatments and subjected to Western blotting, which was performed in accordance with standard protocols. Briefly, the proteins of the U266 cells were extracted on ice using an RIPA buffer (150 mM NaCl, 50 mM Tris-HCl, pH 8, 0.5% sodium deoxycholate, 1% NP-40, 0.1% sodium dodecyl sulfate (SDS)). Total proteins (50 μg) were size-fractionated with SDS/PAGE transferred to a polyvinylidene difluoride membrane, and then blocked for 1 h with 5% skimmed milk. Next, the protein bands were incubated with monoclonal antibodies (SURVIVIN, BCL-XL, cleaved caspase-3/9). Data were normalized using GAPDH. The blots were detected using an enhanced chemiluminescence system.

### Statistical analysis

Data are presented as mean ± standard deviation and analyzed via Student’s t-test on SPSS 23.0 (Chicago, US). *P* < 0.05 was considered statistically significant.

## Results

### Characterization of CXCR4-PEG-CdTe-DOX

**Fig. 2:**
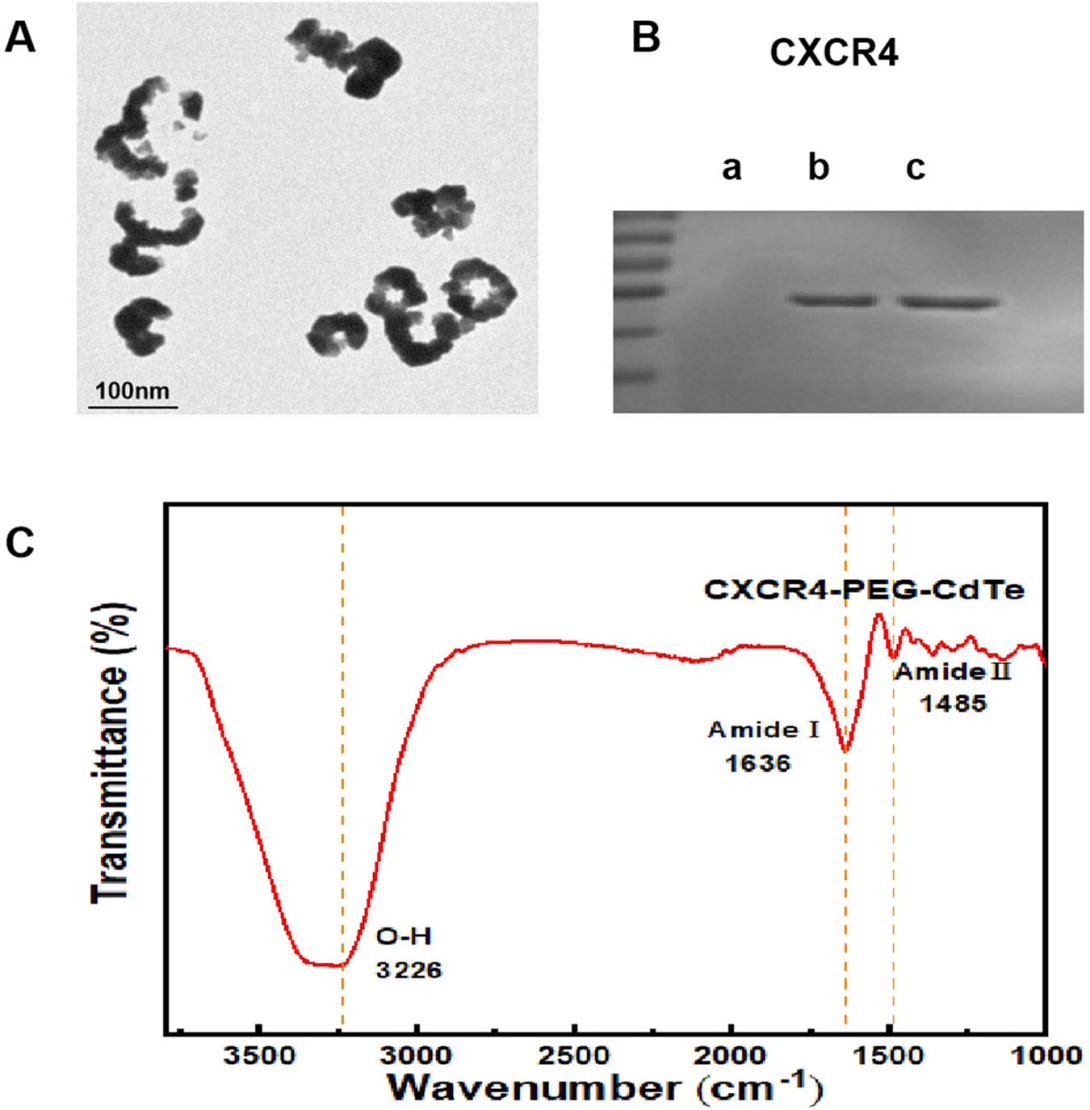
Characterization of nanoloaded drug synthesis. **A** Morphological pattern of PFG-CdTe electron microscope; (**B**) connection rate of CXCR4-PEG-CdTe (b) compared with antiCXCR4Ambs (c) was about 75% under Coomassie brilliant blue staining; (**C**) infrared spectra of CXCR4 and PEG-CdTe connected by an amide linkage

**Fig. 3:**
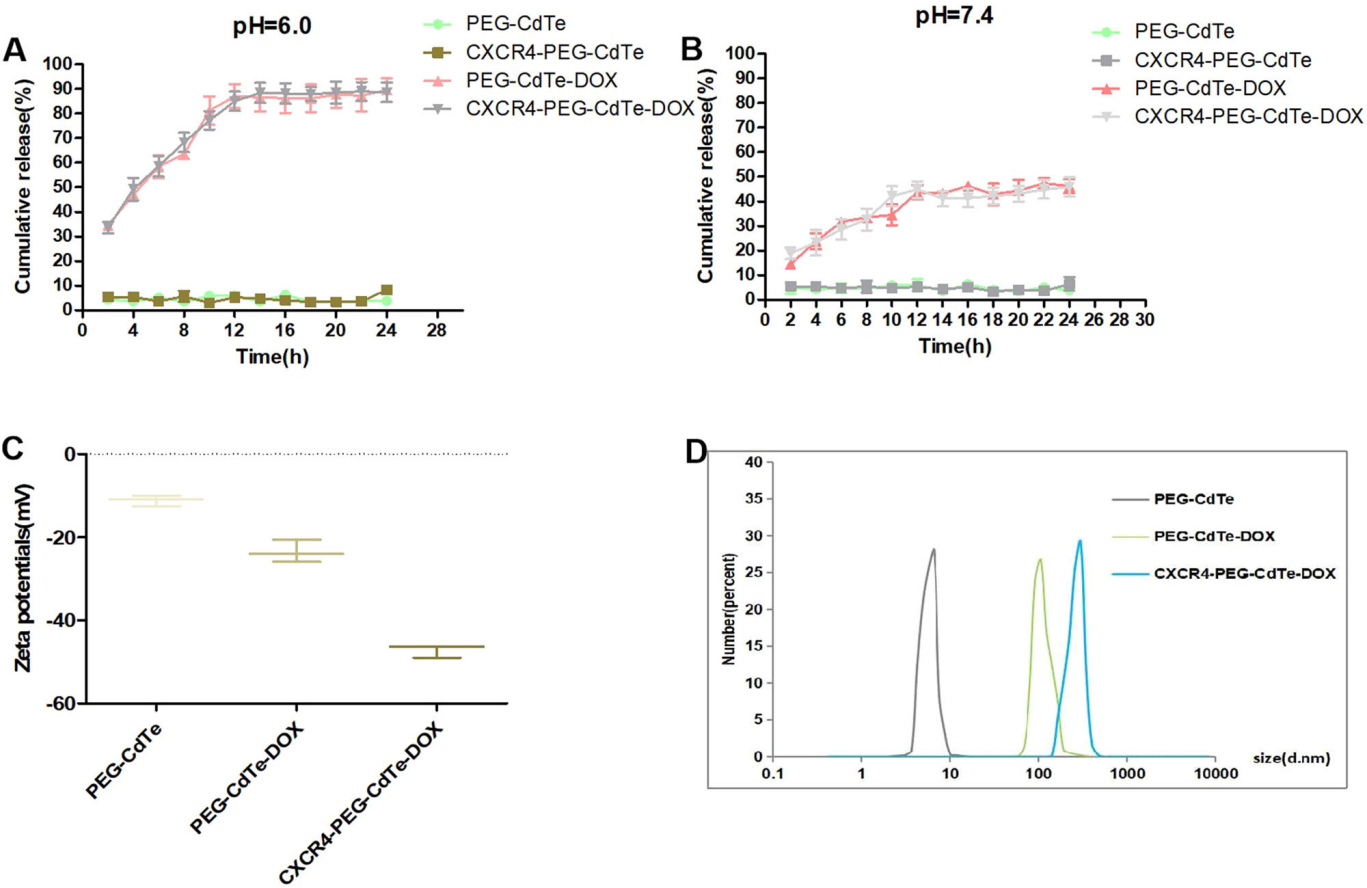
**A**, **B** DOX drug release of CXCR4-PEG-CdTe-DOX at different pHs, suggesting faster drug release in acidic environment; **C**, **D** Zeta potentials, average sizes of PEG-CdTe, PEG-CdTe-DOX and CXCR4-PEG-CdTe-DOX detected with DLS

The PEG-CdTe QDs are spherical particles coated in PEG (Fig. 2A). The Coomassie brilliant blue staining showed that anti-CXCR4 monoclonal antibodies were successfully linked to the PEG-CdTe QDs and the ligation rate was about 75% (Fig. 2B). CXCR4 and PEG-CdTe QDs were substantiated by amide bonds, and corresponded to C = O stretching vibration and O-H bending vibration respectively on the spectrum of CXCR4-PEG-CdTe (1636 cm^−1^ in Fig. 2C). The cumulative release of DOX from CXCR4-PEG-CdTe-DOX was shown in Fig. 3. DOX was released the most rapidly at pH 6.0, where about 90% of the loaded DOX was released within 14 h (Fig. 3A). At pH 7.4, however, the release ratio of the CXCR4-PEG-CdTe-DOX nanoparticles was considerably slower than that at pH 6.0 (Fig. 3B). This finding suggests the release of DOX from CXCR4-PEG-CdTe is pH-triggered. .The average hydrodynamic diameters of the PEG-CdTe nanoparticles, PEG-CdTe-DOX nanoparticles, and CXCR4-PEG-CdTe-DOX were about 8.34 ± 1.028, 110.32 ± 5.375 and 262.52 ± 9.036 nm respectively (Fig. 3D). The Zeta potentials of the three samples were −11.09 ± 1.306, −23.46 ± 2.706 mV and −47.17 ± 1.576 mV respectively (Fig. 3C). DOX (1 mL, 1 mg/mL), PEG-CdTe (1 mL, 1 mg/mL) and anti-CXCR4 monoclonal antibodies (10 uL, 100 ug/mL) were mixed to reach the highest DL and EE. The DL and EE of CXCR4-PEG-CdTe-DOX maximized to 44.29 ± 0.17% and 85.5 ± 0.53% respectively.

### Drug delivery image

**Fig. 4:**
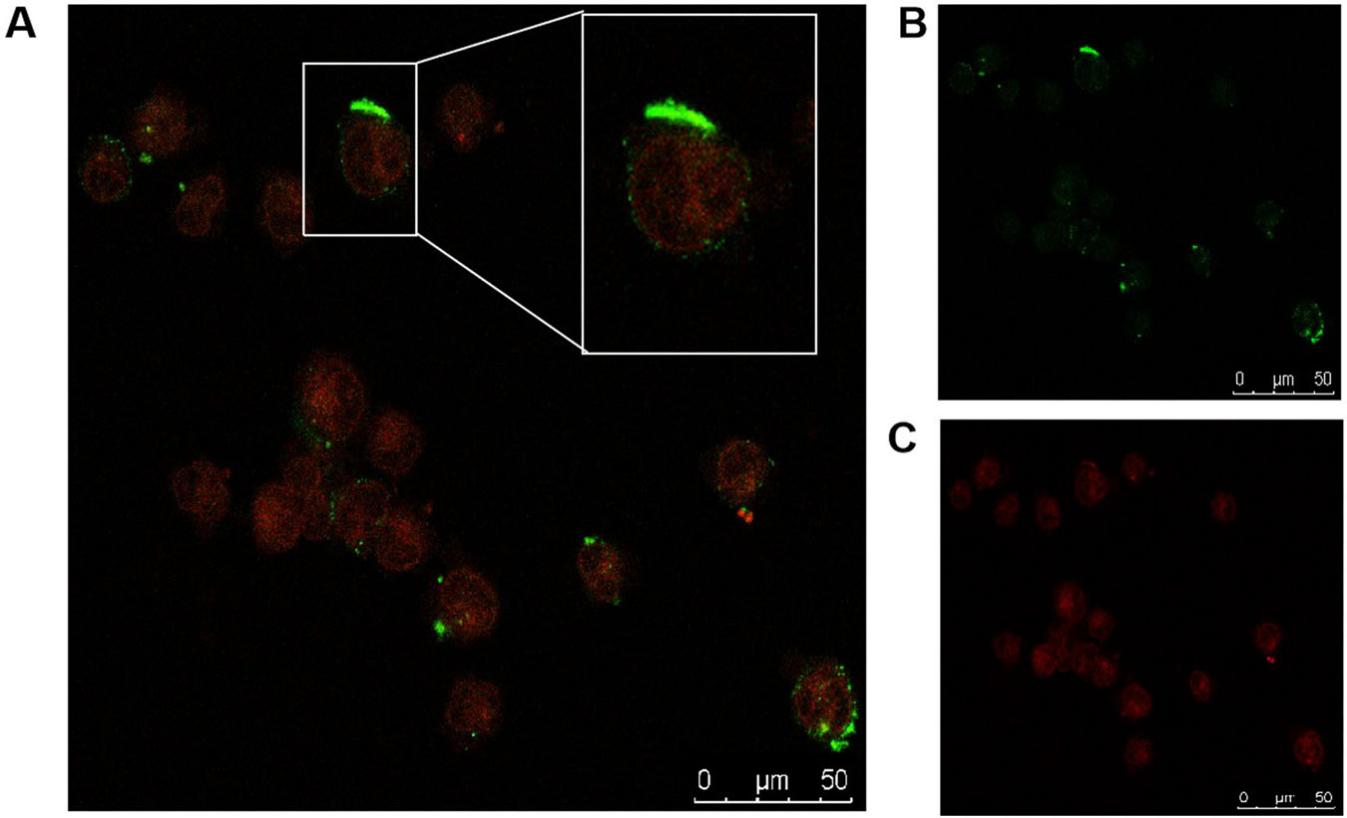
Confocal microscopy showing CXCR4-PEG-CdTe-DOX can targetedly bind to U266 cells and efficiently deliver DOX into the cells (**A**), with FITC-CXCR4 in green (**B**) and DOX in Red (**C**)

**Fig. 5:**
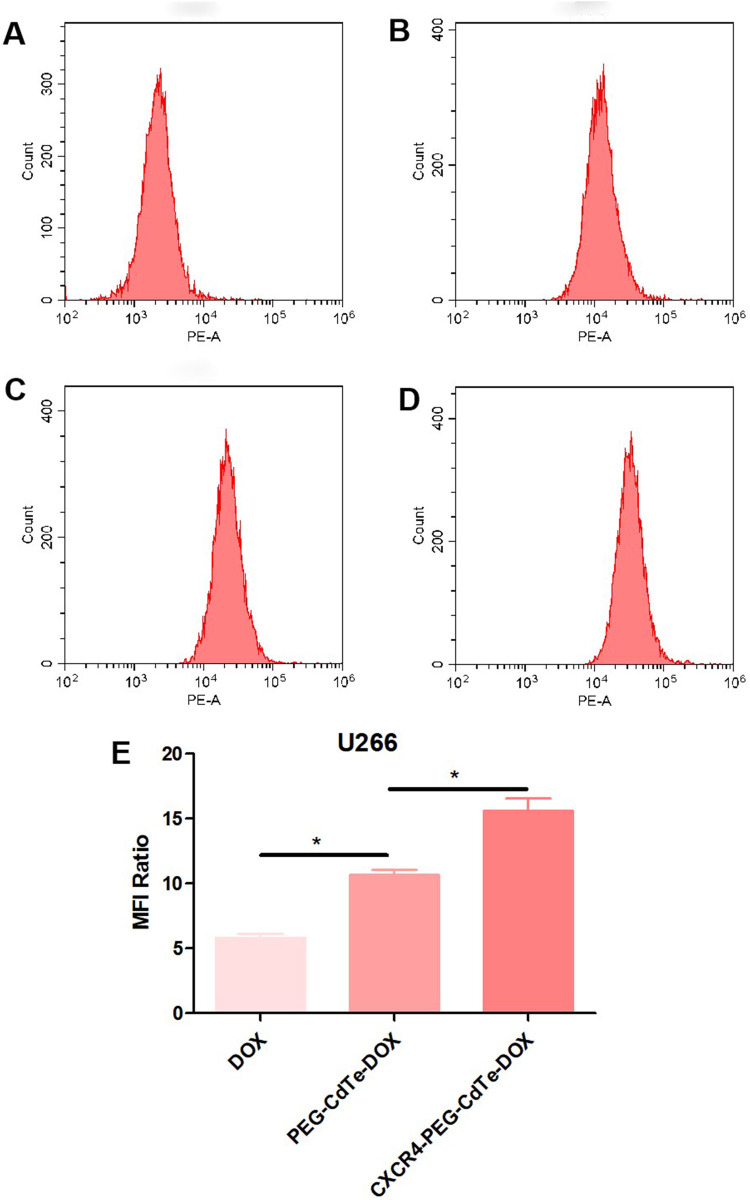
U266 cells were cultured at pH 7.4 with control (**A**), DOX (**B**), PEG-CdTe-DOX (**C**) and CXCR4-PEG-CdTe-DOX (**D**) at the same DOX concentration for 3 h, and drug fluorescence intensity of DOX in U266 was measured by flow cytometry. Drug fluorescence intensity of DOX in the CXCR4-PEG-CdTe-DOX group was significantly increased (**E**) (*P < 0.05)

The fluorescence images under a laser scanning confocal microscope further confirmed that DOX and FITC-CXCR4 were conjugated to the PEG-CdTe (Fig. 4). FITC (green) as the fluorescence probe was loaded to track the nanoparticles, and DOX was labeled with cell nucleus (red). Moreover, the conjugation of FITC-CXCR4 to PEG-CdTe-DOX was verified by the green fluorescence on the surface of the CXCR4-PEG-CdTe-DOX-treated U266 cells, which can enhance intracellular drug concentration through actively targeting tumor cells. The DOX concentration in the U266 cells was monitored through FCM of intracellular fluorescence intensity (Fig. 5). Intracellalar DOX concentration significantly rose after treatment with CXCR4-PEG-CdTe-DOX compared with free DOX or PEG-CdTe-DOX.

### Cytotoxicity assay

**Fig. 6:**
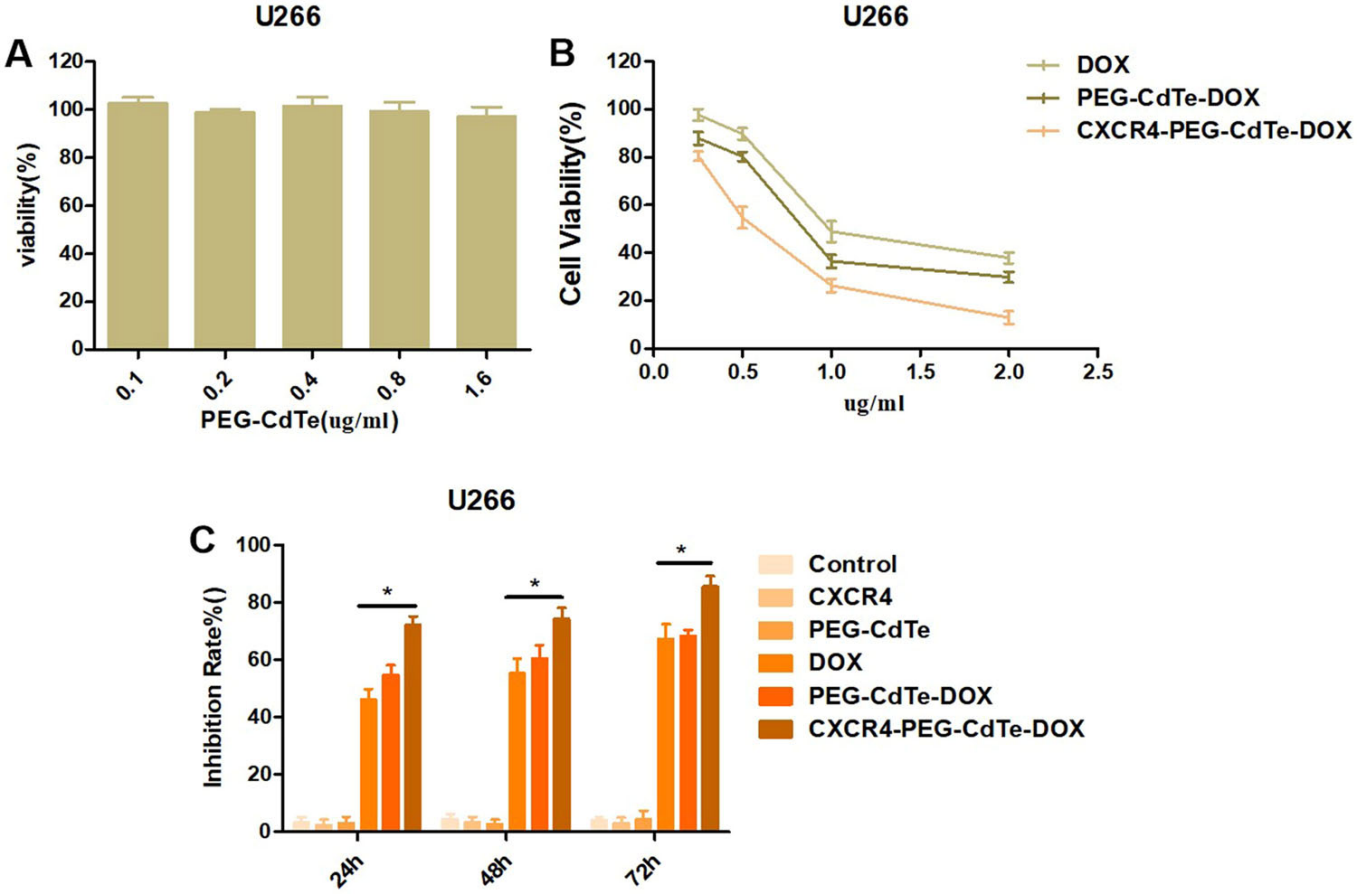
**A** No obvious toxicity to U266 cells in the tested range of PEG-CdTe concentration (0.1–1.6 μg/mL); (**B**) IC_50_ after 24 h in U266 cells (0.979 μg/mL), the PEG-CdTe-DOX group (0.854 μg/mL), and CXCR4-PEG-CdTe-DOX group (0.579 μg/mL); (**C**) control group, CXCR4 group, PEG-CdTe group, DOX group, PEG-CdTe-DOX group and CXCR4-PEG-CdTe-DOX group, with the IC_50_ of DOX, were co-cultured with U266 cells for 24, 48 or 72 h; activity of U266 cells was detected by CCK8; the highest inhibition rate of U266 cells was found in the CXCR4-PEG-CdTe-DOX group

The viability of U266 cells was not inhibited significantly when the PEG-CdTe concentration was 0.1–1.6 ug/mL (*P* > 0.05, Fig. 6A), which indicating its toxicity is low and its concentration is within the tested range. Then the viability of U266 cells treated with DOX, PEG-CdTe-DOX or CXCR4-PEG-CdTe-DOX at different concentrations of DOX (0.5, 1.0, 1.5, 2.0 and 2.5 µg/mL) for 24 h was detected. Next, the cytotoxic effects were tested using CCK-8 assay. The inhibitory concentrations (IC_50_) for DOX, PEG-CdTe-DOX and CXCR4-PEG-CdTe-DOX were 0.979, 0.854, and 0.579 μg/mL, respectively (Fig. 6B). The inhibition rates in U266 cells treated with CXCR4-PEG-CdTe-DOX were 72.2, 74.2 and 85.6% after 24, 48 and 72 h of incubation respectively, which were significantly higher compared with other groups (*P* < 0.05, Fig. 6C).

### Increase in apoptosis by CXCR4-PEG-CdTe-DOX

**Fig. 7:**
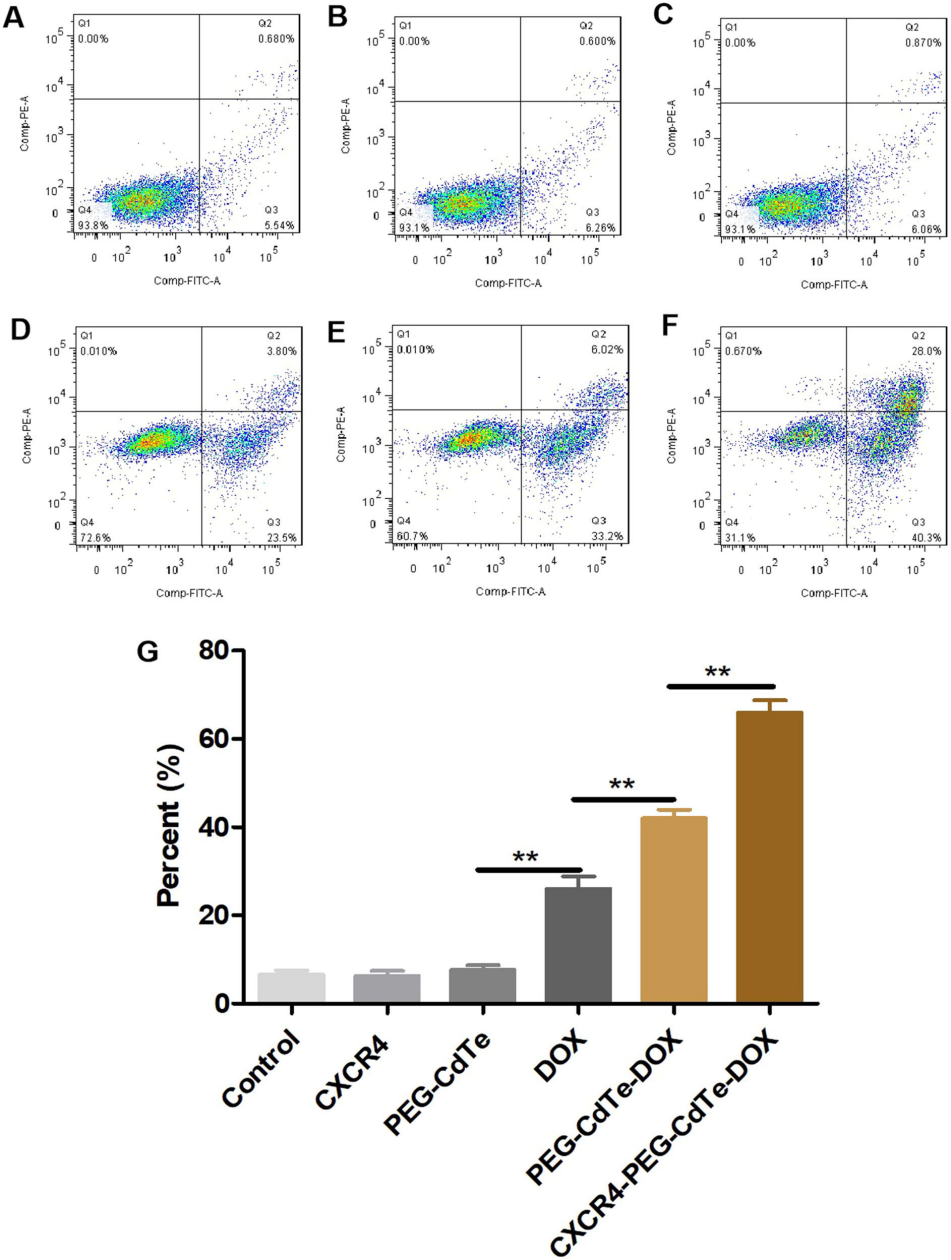
Flow apoptosis of U266 cells. After the control group (**A**), CXCR4 group (**B**), PEG-CdTe group (**C**), DOX group (**D**), PEG-CdTe-DOX group (**E**) and CXCR4-PEG-CdTe-DOX group (**F**) were co-cultured with U266 cells for 24 h, apoptosis rate in the CXCR-PEG-CdTe-DOX group was significantly higher compared with the DOX group and the PEG-CdTe-DOX group (**G**) (**P < 0.01)

U266 cells treated with PBS, CXCR4, PEG-CdTe, DOX, PEG-CdTe-DOX or CXCR4-PEG-CdTe-DOX at 0.979 μg/mL DOX for 24 h were observed via FCM (Fig. 7). The total apoptosis rates were 6.22, 6.86, 9.63, 27.3, 39.22 and 68.3%, respectively. The apoptosis rates were significantly different among the DOX, PEG-CdTe-DOX, and CXCR4-PEG-CdTe-DOX groups. The highest apoptosis rate was observed in the CXCR4-PEG-CdTe-DOX group (*P* < 0.01).

### Mechanisms for CXCR4-PEG-CdTe-DOX-induced apoptosis

**Fig. 8:**
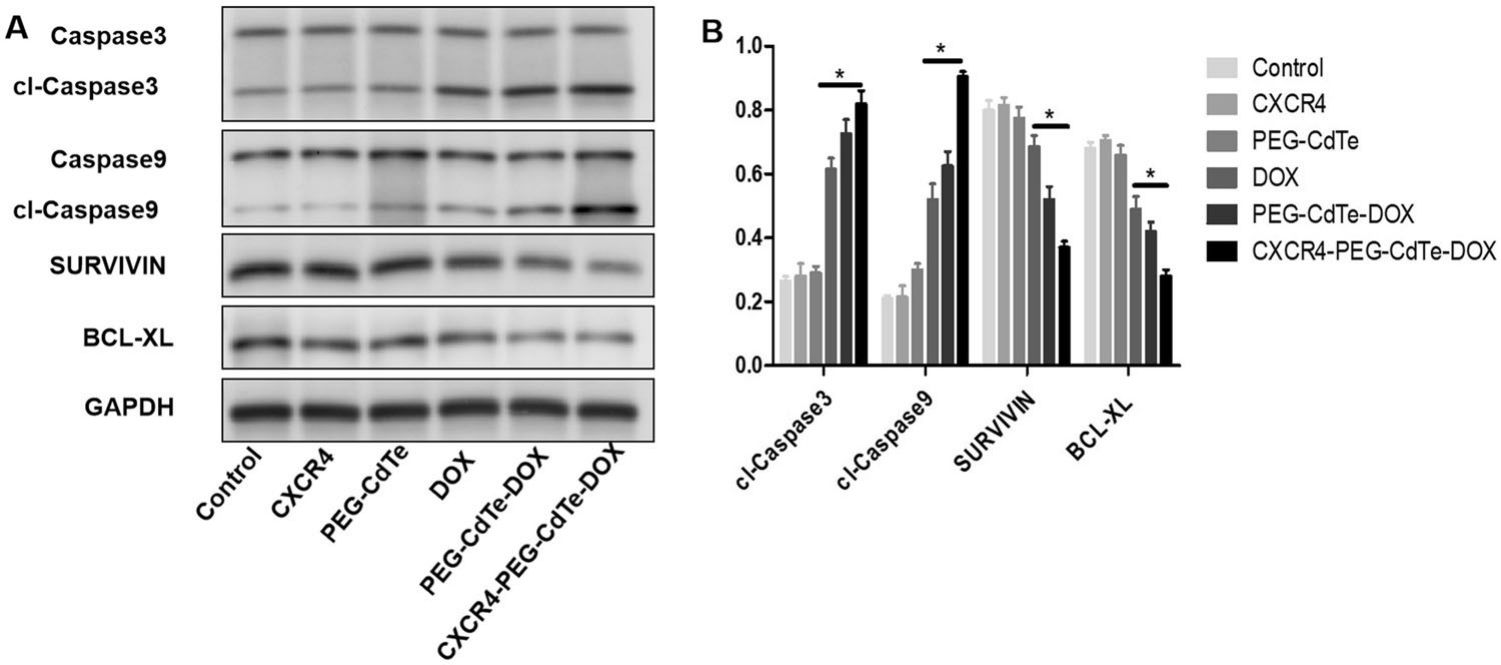
Expressions of apoptotic proteins. After the control group, CXCR4 group, PEG-CdTe group, PEG-CdTe-DOX group and CXCR4-PEG-CdTe-DOX group were co-cultured with U266 cells for 24 h (**A**). Western blotting showed that the expressions of pro-apoptotic cl-caspase 3 and cl-caspase 9 significantly increased and the expressions of apoptotic SURVIVIN and BCL-xl significantly decreased in the CXCR4-PEG-CdTe-DOX group compared with the DOX group (**B**) (*P < 0.05)

The protein expressions of cleaved caspase-9 and -3 were upregulated, while those of BCL-XL and SURVIVIN were downregulated in the DOX, PEG-CdTe-DOX and CXCR4-PEG-CdTe-DOX groups compared with the control (Fig. 8). These changes were particularly significant in the CXCR4-PEG-CdTe-DOX group (*P* < 0.05). The above results indicate that CXCR4-PEG-CdTe-DOX can improve the antitumor activity of DOX.

## Discussion

Currently, the treatment of MM is still dominated by chemotherapy-based therapy, but drug resistance usually leads to treatment failure. DOX induces tumor cell death by inserting base pairs into DNA strands and destroying DNA repairs mediated by topoisomerase II, or by producing free radicals that cause lipid peroxidation and oxidative damage to cell membranes, proteins, and DNA [16]. However, DOX is non-selective, and has abvious toxic and side effects in clinic, including myelosuppression, especially cardiotoxicity. Nevertheless, our study found nanoscale CXCR4-PEG-CdTe-DOX can significantly increase the drug concentration in the targeted MM cells (Fig. 4) [14, 17], thus reducing the toxic side effects on normal tissues. On the one hand, CdTe QDs are a potential risk to organisms because of toxicity. On the other hand, PEG has hydrophilicity, biocompatibility, safety, non-toxicity and low immunogenicity. The conjugation of PEG and CdTe induces an “immune neglect” effect, reducing or even avoiding plasma opsonization and the absorption and phagocytosis of particles by the reticular endothelial system [18]. This property allows PEG to prolong the blood circulation time of particles by minimizing or eliminating the adsorption of plasma proteins by these particles [19]. PEG-modified CdTe nanoparticles are efficiently internalized by the cells through fluid-phase endocytosis as well as the affinity of the lipid bilayer to the plasma membrane surface [20]. The above nanocarriers has (1) high drug-loading capacity, a wide range of drugs, (2) slow release, prolonged action time of drugs, (3) small particle size, narrow and unique distribution through physiological barriers, (4) modifies the hydrophilic end to prevent protein adsorption and avoid the capture by the reticular endothelial system, (5) connects the target to actively reach the target site, (6) reduces the drug dose while ensuring efficacy to reduce or avoid side effects [21–23]. Therefore, CXCR4-PEG-CdTe is an effective and safe active targeting vector.

Apoptosis is a programmed cell death that leads to regular and efficient elimination of injured cells, such as the cell death due to DNA damage or amid growth. Apoptosis can be started by internal signals (e.g., genotoxic stress) or extrinsic signals (e.g., connection of ligands to cell surface death receptors) [24]. Bcl-xL plays both canonical roles (e.g., pro-survival, avoidance of apoptosis, induced drug resistance) and non-canonical roles (e.g., improved cell migration and invasion, angiogenesis) [25]. Antiapoptotic proteins (Bcl-2, Bcl-xL) keep from the disruption of mitochondrial outer membrane permeability (MOMP) and thus prevent mitochondrial- dependent death in the cytosol [26]. Similarly in our study, the Bcl-xl expression was lower when more apoptosis occurred SURVIVIN is a small protein with different isoforms, which mostly are related to inhibited apoptosis and promoted cell proliferation [27]. SURVIVIN expression is usually associated with the poor prognosis, disease progression, and drug resistance of myeloma patients [28]. Caspases are central to the mechanism of apoptosis as they are both the initiators (e.g., caspase-2, -8, -9 and -10, which are primarily responsible for the beginning of the apoptotic pathway) and executors (e.g., caspase-3, -6 and -7, which are responsible for definite cleavage of cellular components) of cell death [29]. Our study revealed that the protein expressions of SURVIVIN and cleaved caspase-3/9 decreased and increased respectively after the DOX treatment in vitro. The difference was more obvious in the CXCR4-PEG-CdTe-DOX group, indicating CXCR4-PEG-CdTe-DOX is an effective delivery vehicle.

In summary, we report a novel delivery vehicle that promotes anti-tumor effects. CXCR4-PEG-CdTe-DOX can proactively target CXCR4 antigen on the surface of U266 cells, and can effectively transport DOX to tumor cells. Based on these results, we propose to use CXCR4-PEG-CdTe-DOX nanoparticles as the optimal delivery system for EMM.

## Data Availability

All available data and material are original work. All data have been clearly provided in the manuscript without additional data or supporting material. The datasets used and analyzed in the current study are also available from the corresponding author on reasonable request.
